# Magnetic resonance imaging (MRI) for local staging before salvage radical prostatectomy: a meta-analysis

**DOI:** 10.1007/s00345-023-04383-2

**Published:** 2023-04-05

**Authors:** Piotr Zapała, Aleksander Ślusarczyk, Paweł Rajwa, Giorgio Gandaglia, Łukasz Zapała, Fabio Zattoni, Tomasz Lorenc, Guillaume Ploussard, Piotr Radziszewski

**Affiliations:** 1 Clinic of General, Oncological and Functional Urology, Medical University of Warsaw, Lindleya 4, 02-005 Warsaw, Poland; 2 Department of Urology, Medical University of Vienna, Vienna, Austria; 3 Department of Urology, Medical University of Silesia, Zabrze, Poland; 4 Unit of Urology/Division of Oncology, IRCCS San Raffaele, San Raffaele Hospital, Milan, Italy; 5 Department of Surgery, Oncology and Gastroenterology, University of Padua, 35128 Padua, Italy; 6 1St Department of Clinical Radiology, Medical University of Warsaw, Warsaw, Poland; 7 Department of Urology, La Croix du Sud Hospital, Quint Fonsegrives, France

**Keywords:** Prostate cancer, Magnetic resonance, MRI, Radiorecurrent, Salvage prostatectomy

## Abstract

**Purpose:**

The reliability of magnetic resonance imaging (MRI) as a local and nodal staging tool in radio-recurrent prostate cancer (PCa) is still unclear. The present study aims at evaluating the predictive value of MRI in the detection of extracapsular extension (ECE), seminal vesical invasion (SVI) and nodal involvement (LNI) in patients after primary radio (EBRT) and/or brachytherapy (BT) before salvage radical prostatectomy (SRP).

**Methods:**

This systematic review and meta-analysis were performed in line with the Preferred Reporting Items for Systematic Reviews and Meta-analyses (PRISMA) guidelines. Pubmed, Scopus, and Web of Science databases were systemically reviewed to extract the data on diagnostic performance of MRI in radio-recurrent PCa.

**Results:**

Four studies comprising 94 radio-recurrent PCa patients were included. The pooled prevalence of ECE, SVI, and LNI was 61%, 41%, and 20%, respectively. The pooled sensitivity for ECE, SVI and LNI detection was 53% (CI 95% 19.8–83.6%), 53% (CI 95% 37.2–68%) and 33% (CI 95% 4.7–83.1%) respectively, whereas specificity was 75% (CI 95% 40.6–92.6%), 88% (CI 95% 71.7–95.9%) and 92% (CI 95% 79.6–96.8%). The sensitivity analysis revealed that a single outlying study using only T2-weighted imaging instead of multiparametric MRI reported significantly higher sensitivity with significantly lower specificity.

**Conclusions:**

This is the first meta-analysis reporting reliability of staging MRI in a radio-recurrent setting. MRI provides poor sensitivity while maintaining high specificity for local and nodal staging before SRP. However, current evidence is limited to the low number of heterogenous studies at meaningful risk of bias.

**Supplementary Information:**

The online version contains supplementary material available at 10.1007/s00345-023-04383-2.

## Introduction

In patients with non-metastatic prostate cancer (PCa) radiation therapy (RT), and brachytherapy (BT) can substitute radical surgery as treatment with curative intent in the primary disease setting. The 9-year cancer control rate for external beam radiotherapy (EBRT) in localized disease is estimated to be 50–80% depending on the risk group [1]. Approximately 10 to 40% of patients will recur biochemically after permanent BT during 12 years of follow-up [2]. Patients who fail primary radiotherapy because of local recurrence can be candidates for local salvage therapy, which might spare or postpone systemic treatment. Exclusion of distant metastases in patients who recur biochemically can be early achieved with positron emission tomography (PET) in particular when utilizing novel radiotracers like 68 Ga-PSMA [3]. However, the value of PET-CT is mainly proven for recurrent lesion localization since its value in local staging remains limited due to poor spatial resolution. In this setting magnetic resonance imaging (MRI) constitutes the most useful imaging modality. In the treatment-naïve cohorts, multiparametric MRI (mpMRI) has shown heterogenous T-staging performance with pooled sensitivity and specificity of 61% and 88% respectively in the overall T3 assessment [4]. Assessment of irradiated gland is, however, associated with certain tissue alterations [5] which might affect the assessment of MRI and might require multiparametric protocol using dynamic contrast-enhanced sequences. The evidence on the reliability of MRI in patients after primary radiation and/or brachytherapy is limited to several studies using biopsy as a reference, which cannot accurately reflect the multifocality and the exact extent of recurrent, aggressive lesions [6–9]. Since biopsy provides limited and indirect insight into local pathological advancement these studies cannot be used for the validation of MRI as a staging tool. To the best of our knowledge, to date, only four studies have used whole-mount specimens as references for the evaluation of MRI staging performance [10–13] before salvage radical prostatectomy (SRP).

We aimed to perform a systematic review and meta-analysis summarizing existing evidence on the predictive value of MRI in assessing extracapsular extension (ECE), seminal vesicle involvement (SVI) and lymph node involvement (LNI) in radio-recurrent prostate cancer patients who are candidates for salvage prostatectomy.

## Materials and methods

### Search strategy

The study was registered with the International prospective register of systematic reviews PROSPERO (ID: CRD42022359818). A systemic literature review was performed in line with the Preferred Reporting Items for Systematic Reviews and Meta-analyses statement. We queried Medline (Pubmed), Scopus and Web of Science databases on September 2022. The search terms included the following: “MRI”, “magnetic resonance imaging”, “prostate cancer”, “radiotherapy” and “salvage prostatectomy”. Two investigators (PZ and AŚ) performed an independent initial screening based on the titles and abstracts. The causes of the exclusion of ineligible reports were noted. Full texts were retrieved and evaluated for eligibility. In case of discrepancies, disagreements were solved by the authors’ consensus.

### Study selection

We included studies analyzing patients with radio-recurrent, clinically non-metastatic PCa managed with salvage prostatectomy (population) who underwent preoperative prostate MRI with detected ECE, SVI and LNI (intervention) compared with patients without the following features (comparison). We analyzed the diagnostic performance of MRI detecting ECE, SVI and LNI (outcome) in prospective and retrospective studies (study design). Only studies using postprostatectomy (whole-mount) specimens as references were considered eligible. Studies were included if they provided true positives (TPs) defined as the presence of both radiological and pathological ECE, SVI or LNI, true negatives (TNs) defined as lack of radiological suspicion of ECE, SVI or LNI in patients without corresponding pathological feature in the postprostatectomy specimen, false positives (FPs) defined as radiological suspicion of ECE, SVI or LNI in patients without corresponding pathological feature in the postprostatectomy specimen and false negatives (FNs) defined as lack of radiological suspicion of ECE, SVI or LNI in patients with presence of the corresponding pathological feature in the postprostatectomy specimen. Reviews, meta-analyses, letters, editorials, meeting abstracts, case reports, and non-English articles were excluded. In the case of duplicate cohorts, the study with more robust data were selected. The references of manuscripts considered eligible were also screened for additional studies.

### Data extraction

Two reviewers (PZ and AŚ) separately extracted data on the study including the author’s name, publication year, number of patients, radiotherapy modality, use of endorectal coil (ERC), MRI modalities sequences (T2, DCI, DCE), MRI protocol used, Prostate Imaging Reporting and Data System (PIRADS) use, the experience of a radiologist, type of MRI scanner (1.5 T vs 3 T), previous hormonotherapy as well as the number of TP, FP, FN, and TN for the main outcomes (ECE, SVI and LNI). Extraction discrepancies were resolved by the authors’ consensus.

### Risk of bias and applicability

Included studies were analyzed for risk of bias and applicability with the revised Quality Assessment of Diagnostic Accuracy Studies tool (QUADAS-2). The index test was defined as the staging MRI of the prostate. Pathological staging based on the whole-mount specimen was used as a reference.

### Statistical analysis

All data were analyzed using R version 4.0 (2020; R Foundation for Statistical Computing, Vienna, Austria). Statistical significance was set at *p* < 0.05. Pooled sensitivity, specificity, positive predictive value (PPV), negative predictive value (NPV), and diagnostics odds ratio (DOR) were calculated and supplemented with forest plots with 95% confidence intervals (CI). For heterogeneity evaluation, the Cochrane *Q* test and the *I*^2^ test were used with significant heterogeneity indicated by *p* < 0.05 in the Cochrane *Q* tests and *I*^2^ > 50%. The sensitivity analysis included separate analysis of studies utilizing mpMRI [10, 12, 13] and the study where only T2-weighted MRI was used [11].

## Evidence synthesis

### Study selection and characteristics

**Fig. 1:**
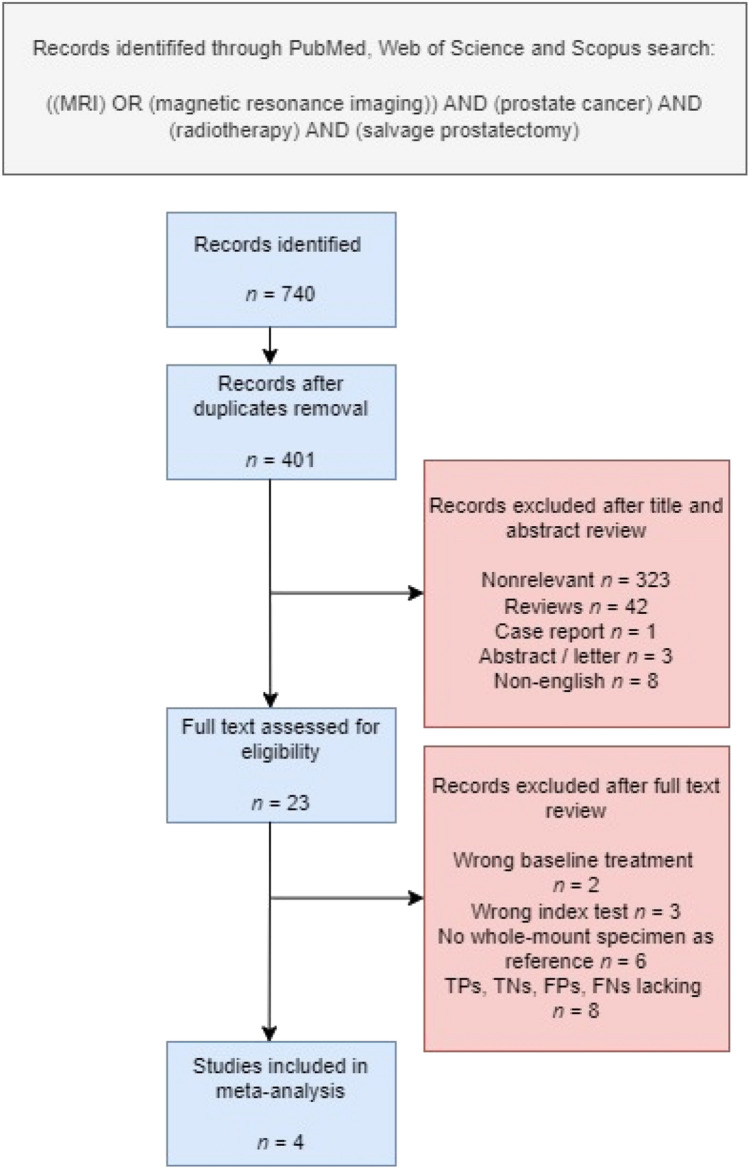
PRISMA flowchart. MRI magnetic resonance imaging, TPs true positives, TNs true negatives, FPs false positives, FNs false negatives

The PRISMA flowchart is depicted in Fig. 1. A total of 4 studies with 94 patients were included (Table 1A) [10–13]. All studies were single-center and retrospective. Imaging reviews were performed centrally by two independent genitourinary MRI radiologists in three studies [10–12] whereas in 1 study review path was not specified [13]. The prevalence of ECE ranged from 50% [13] to 87.5% [12] and the prevalence of SVI ranged from 33.3% [13] to 68.42% [10]. Nodal involvement was reported in 26.32% and 17.78% of patients in the studies by Zattoni and Sala, respectively [10, 11].

**Table 1:** Baseline characteristics with adverse features’ frequencies (A) and type of primary treatment, MRI assessment and systemic therapy before salvage prostatectomy in analyzed studies (B)

A							
Author (references)	Year	Country	Study period	Number of patients	ECE (*n*, %)	SVI (*n*, %)	LNI (*n*, %)
Zattoni [10]	2017	USA	2007–2014	19	14 (73.68%)	13 (68.42%)	5 (26.32%)
Sala [11]	2006	USA	1998–2004	45	19 (42.22%)	19 (42.22%)	8 (17.78%)
Kowa [12]	2021	UK	2014–2017	24	21 (87.5%)	11 (45.83%)	Not provided
Sutani [13]	2019	Japan	2010–2017	6	3 (50%)	2 (33.33%)	Not provided

B							
Author (references)	Primary treatment	MRI field	MRI sequences	Endorectal coil use	PIRADS use	HT or CHT before SRP	Time from MRI to SRP (days; median, IQR)
Zattoni [10]	EBRT and/or BT	3.0 Tesla	T1, T2, DCE, DWI	Yes, routinely	Yes	Yes (21%)	137.5 (84.5–177.3)
Sala [11]	EBRT and/or BT	1.5 Tesla	T1, T2	Yes, routinely	No	Yes (32%)	154 (95.2–179.2)
Kowa [12]	EBRT	1.5 or 3.0 Tesla	T1, T2, DCE, DWI	Yes (16.67%)	Yes	N/R	N/R
Sutani [13]	EBRT	N/R	T1, T2, DCE, DWI	N/R	N/R	No	N/R

ECE extracapsular extension, SVI seminal vesicle invasion, LNI lymph node involvement, USA United States of America, UK United Kingdom, MRI magnetic resonance imaging, PIRADS Prostate Imaging Reporting and Data System, HT hormonal therapy, CHT chemotherapy, SRP salvage radical prostatectomy, IQR interquartile range, EBRT external beam radiotherapy, BT brachytherapy, DCE dynamic contrast-enhanced imaging, DWI diffuse-weighted imaging, N/R not reported

Two studies enrolled patients after EBRT and/ or BT as primary treatment [10, 11] whereas in the remaining two [12, 13] all patients recurred after EBRT. MRI sequences, as well as the MRI field and endorectal coil use, differed between the studies. In the study by Zattoni et al. 3.0 Tesla field was used [10], in the study by Sala et al. 1.5 Tesla field was used [11], whereas Kowa et al. used a 1.5 T field in two patients (8.33%) and a 3.0 Tesla field in the remaining twenty-two (91.67%) [12]. In the study by Sutani [13] data regarding the MRI field utilized was lacking. All the studies [10, 12, 13] except for the study by Sala et al. [11] used T1- and T2-weighted sequences supplemented with dynamic contrast-enhanced imaging and diffusion-weighted imaging. In the study by Sala et al. only T1- and T2-weighted imaging (T1-WI, T2-WI) was used [11]. In the studies by Sala and Zattoni endorectal coil was used routinely [10, 11], whereas in the study by Kowa et al. it was used in four patients (16.67%) [12]. The use of an endorectal coil was not specified in the study by Sutani et al. [13]. Two studies used PIRADS for the evaluation of MRI images [10, 12] whereas the study by Sala et al. was published before the release of PIRADS recommendations [11] and in the study by Sutani it was not specified [13]. The median time interval between staging MRI and SRP was 137.5 (IQR 84.5–177.3) and 154 days (IQR 95.2–179.2) in studies by Zattoni and Sala respectively [10, 11], whereas it was not reported in the remaining studies [12, 13]. In the studies by Zattoni and Sala surgery was performed in a retropubic approach [10, 11] and in the studies by Kowa and Sutani it was performed in a robot-assisted approach [12, 13]. Differences between primary treatment, MRI and prostatectomy are depicted in Table 1B.

The risk of bias and applicability concerns are presented in Supplementary Fig. 1. Due to the use of systemic treatment before SRP after MRI assessment which might result in pathological downstaging, the risk of bias of flow and timing was generally high. The study by Sutani et al. provided a poor description of confounders regarding the index test and the reference which limited bias evaluation, whereas the lack of data on systemic treatment in the study by Kowa impacted flow and timing evaluation.

### Meta-analysis

#### MRI for detection of extracapsular extension

**Fig. 2:**
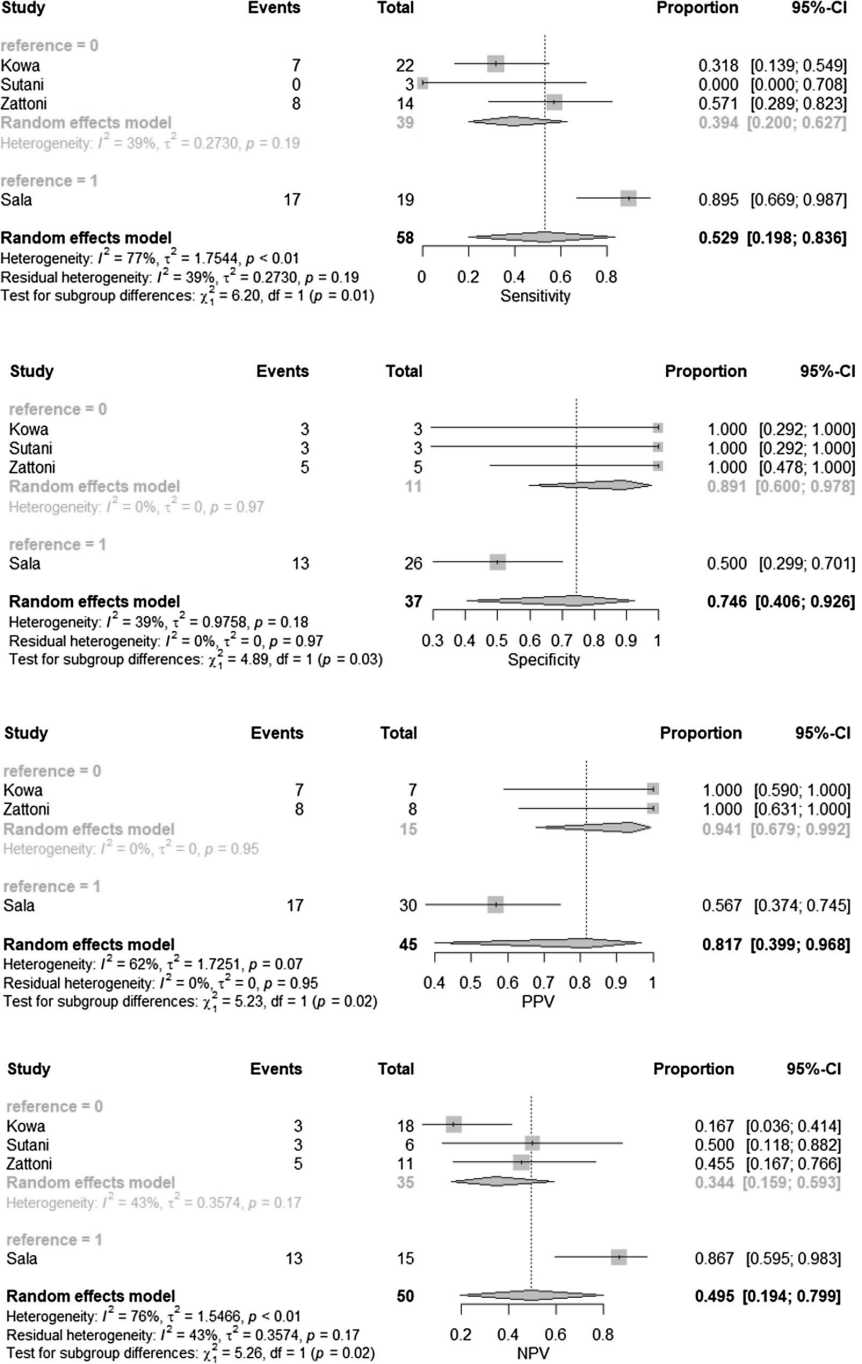
Forest plots for pooled accuracy, sensitivity, specificity, NPV and PPV of extracapsular extension detection including sensitivity analysis for multiparametric MRI (reference = 0) and T2-weighted imaging only (reference = 1)

The pooled ECE prevalence was 61%. There was significant heterogeneity between included studies. Pooled sensitivity, specificity, PPV and NPV were 53%, 75%, 81.7% and 49.5% respectively. Forest plots are depicted in Fig. 2. The pooled DOR was 7.92 (95% CI 2.12–29.58). Dot plot illustrating the association of sensitivity and false positive rate of included studies is depicted in Supplementary Fig. 2.

#### MRI for detection of seminal vesicle invasion

**Fig. 3:**
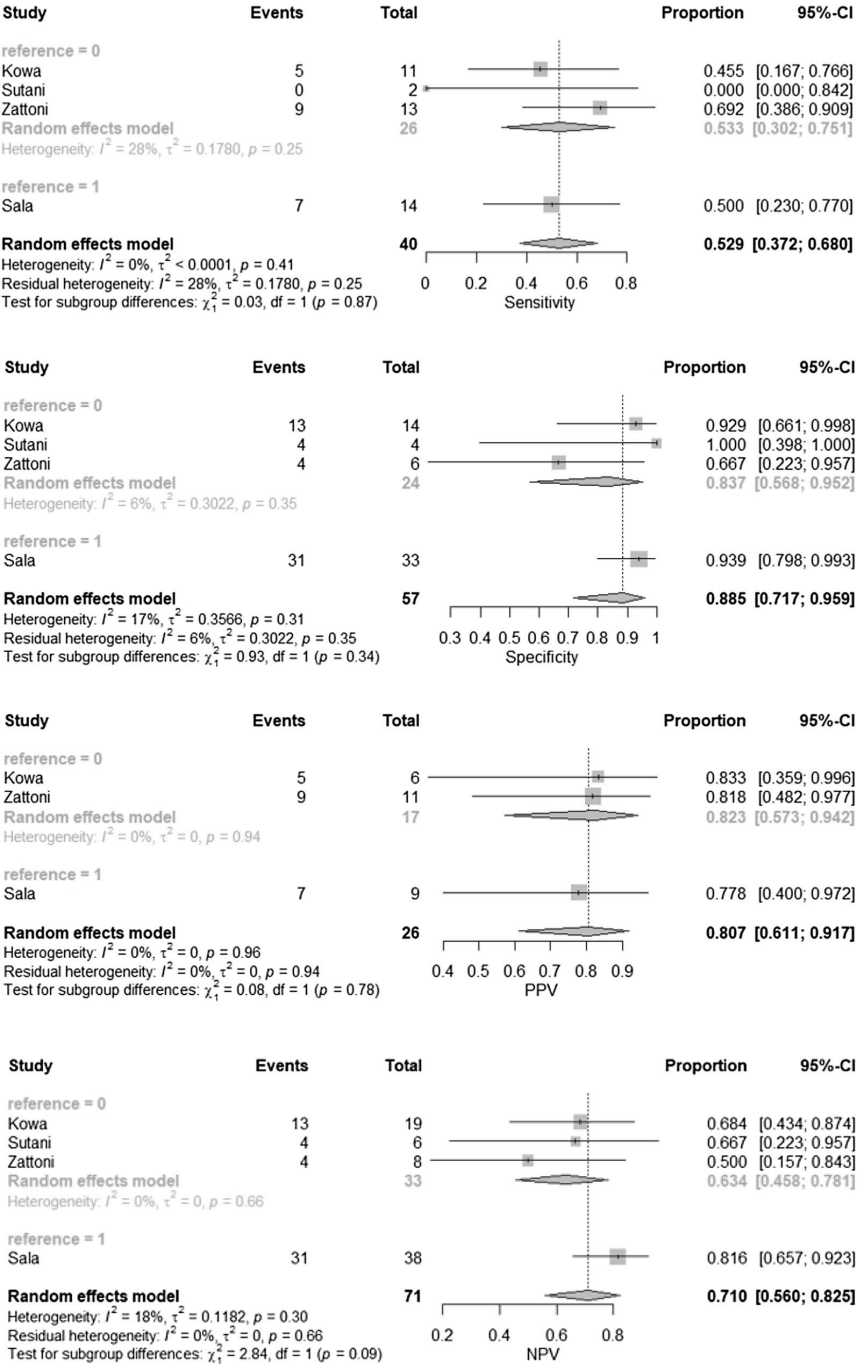
Forest plots for pooled accuracy, sensitivity, specificity, PPV and NPV of seminal vesicle invasion detection including sensitivity analysis for multiparametric MRI (reference = 0) and T2-weighted imaging only (reference = 1)

The pooled SVI prevalence was 41%. There was no significant heterogeneity between included studies. Pooled sensitivity, specificity, PPV and NPV were 52.9%, 88.5%, 80.7% and 71% respectively. Forest plots are depicted in Fig. 3. The pooled DOR was 9.56 (95% CI 2.97–30.74). Dot plot illustrating the association of sensitivity and false positive rate of included studies is depicted in Supplementary Fig. 3.

#### MRI for detection of lymph node involvement

The pooled LNI prevalence was 20%. There was no significant heterogeneity between included studies. Pooled sensitivity, specificity, PPV and NPV were 33.1%, 91.6%, 50.3% and 84.2% respectively. Forest plots are depicted in supplementary Fig. 4. The pooled DOR was 5.23 (95% CI 0.94–29.04). Dot plot illustrating the association of sensitivity and false positive rate of included studies is depicted in Supplementary Fig. 5.

#### Sensitivity analysis: T2-weighted imaging MRI vs multiparametric MRI

To take into account, the lack of functional imaging in the study of Sala et al. we decided to perform a sensitivity analysis. After excluding the study by Sala et al. from analysis, sensitivity, specificity, PPV and NPV were 39.4%, 89.1%, 94.1% and 34.4% for ECE whereas 52.9%, 83.7%, 82.3% and 63.4% for SVI respectively. In the detection of ECE mpMRI [10, 12, 13] revealed higher pooled specificity (*p* = 0.03) and PPV (*p* = 0.02) but lower sensitivity (*p* = 0.01) and NPV (*p* = 0.02) than T2-WI [11].

## Discussion

We present the first meta-analysis on the accuracy of magnetic resonance imaging in local staging of prostate cancer after primary radiation, based on final pathology in RP specimens. The outcomes of our systematic review emphasize the limited evidence on the diagnostic performance of MRI in the radio-recurrent setting. The analysis of included studies indicates that MRI used as the standalone local staging tool has low sensitivity in screening for adverse pathological features after radiation which might undermine its utility in qualification to focal salvage therapy as well as guiding salvage surgery. Simultaneously we observed relatively high specificity of MRI when predicting ECE and SVI which indicates low harm of T3 overdiagnosis. Results of our sensitivity analysis suggest that supplementing T1- and T2-weighted sequences with DWI and/or DCE can be necessary for maintaining specificity. Unexpectedly, additional sequences might not be however incremental in terms of sensitivity or even increase the harm of false negatives when detecting extraprostatic disease.

Several key confounders result in high heterogeneity in included studies. The variety of MRI sequences utilized and PIRADS implementation seem to be crucial methodological discrepancies. The analysis by Sala et al. was the first one on MRI utility in a radio-recurrent setting and it was delivered in the pre-PIRADS era [11]. The staging evaluation consisted basically of T2-WI only. Based on the initial data derived from treatment-naive cohorts ECE detection was suggested to be significantly improved when using T2-WI combined with DCE [14] although the benefit of supplementing T2-WI + DWI with DCE has not been confirmed in the further meta-analysis [15]. In a radio-recurrent setting, DCE is however considered superior to T2-WI. When localizing foci in patients recurring biochemically after EBRT DCE performed significantly better in terms of sensitivity, PPV and NPV while maintaining specificity [16]. This was initially attributed to the confounding radiological presentation of post-radiation fibrosis in T2-WI, which can be however easily distinguished when observing early enhancement typical for hypervascular tumor lesions. Different observational studies suggested that DCE might be inferior to DWI when supplementing T2-WI yielding an accuracy of 0.79–0.86 [17]. Surprisingly, sensitivity analysis evaluating additional functional imaging [10, 12, 13] yielded lower sensitivity and NPV compared to the sole T2-weighted imaging used by Sala [11]. It should be noted that the baseline assessment in the study by Sala included a 5-point scale evaluation with 3, 4 or 5 points assumed to indicate the presence of pathological features at the patient level. In turn, the sensitivities calculated for a higher suspicion threshold (4 or 5 points) on a lesion level were 64% and 39%, respectively, depending on the experience of the radiologist. Accordingly, specificities calculated for ECE detection using a lower cutoff on a patient level were strikingly lower (50% and 46%) than those achieved with a higher cutoff on a lesion level (91% and 86%). Therefore, although sensitivities and specificities reported by Sala at the patient level differed from those in the remaining included studies, outcomes of less conservative, lesion-level analysis seem to correspond with them. The significant accuracy gap between the two cutoffs and analysis levels emphasizes the impact of “language spoken” when assessing and reporting adverse pathology on MRI. The reporting system (PIRADS, Likert or other) and cut-off triggering clinical decisions requires, however, further validation. To reduce the variability of acquisition and reporting in recurrent PCa Prostate Imaging for Recurrence Reporting (PI-RR) was introduced [18]. External, multi-reader validation of PI-RR system in the post-radiation setting has yielded satisfactory accuracy with AUC ranging from 0.77 to 0.92 for detection of recurrence [19], it was however not standardized for local and nodal restaging. Finally, from the practical point of view, what might also improve imaging performance is a comparison between pre- and post-radiotherapy MRI in a single-center setting, preferably by the same genitourinary radiologist.

Two of the studies included patients routinely staged utilizing an endorectal coil [10, 11]. In turn, in the study by Kowa et al. endorectal coil was used only 16.67% of patients [12] whereas the study by Sutani et al. has not reported the use of an endorectal coil [13]. The use of ERC in T2-weighted imaging for staging has been previously introduced as a superior diagnostic alternative to the body-array coil due to a higher signal-to-noise ratio. In the treatment-naïve setting use of ERC increased the sensitivity of ECE detection from 7% to a range of 73–80% while maintaining a specificity of 100% [20]. Simultaneous use of endorectal and external coil was reported to improve staging accuracy from 59 to 78–79% [21]. When projecting these data onto our data, it can be therefore expected that lack of routine ERC use might result in underestimating diagnostic accuracy, especially the ECE detection rate. This seems valid when analyzing our outcomes in a study-by-study approach. Although sensitivity analysis could not be performed due to the heterogeneity of the cohort delivered by Kowa et al. [12], cohorts by Zattoni and Sala, which enrolled ERC MRI-staged patients yielded the two highest sensitivity values for ECE detection [10, 11]. It should be, however, noted that extensive evidence from treatment-naïve setting meta-analysis emphasizes heterogeneous T3-staging sensitivity and at best limited benefit of ERC use [4].

What can greatly impact MRI staging outcome is androgen deprivation prior to SRP which constituted uncontrolled confounder in 2 analyzed studies [10, 11] and potential confounder in 1 study [12]. In the primary, high-risk PCa patients treated with enzalutamide and conventional androgen deprivation therapy more than 90% of lesions demonstrated > 50% volume reduction in posttreatment MRI with almost 40% of patients bearing minimal residual disease only in a postprostatectomy specimen [22]. The effects of neoadjuvant ADT in a primary setting include downstaging as well as lower incidence of positive surgical margins and nodal involvement [23] although evidence on long-term outcome is lacking.

The outcomes of our analysis suggest that the performance of MRI in radio-recurrent patients might be comparable to this achieved in treatment-naïve cohorts before primary RP. Meta-analysis of MRI T-staging utility in treatment-naïve patients yielded poor sensitivity (61%; CI 95% 54–67%) but high specificity (88%; CI 95% 85–91%) when detecting the overall T3 stage [4]. The major restriction of T3 detection arises from the limited sensitivity of T3a prediction, which often presents as microscopic ECE. Notably, the specificities of ECE, SVI and LNI detection in the primary staging setting are generally high (91%, 96% and 88% respectively) which corresponds with our results [4]. In the light of MRI limitations, the potential role of additional imaging including novel PET-CT modalities is increasing. In a radio-recurrent setting, the fundamental role of PET imaging is a primary tool of metastatic screening, however, PET-CT might be also a valuable supplement to MRI with a combined detection rate of relapse site exceeding 70% [6]. Since the spatial resolution of PET-CT is being constantly improved, there is a strong rationale for the head-to-head comparison. Finally, promising pooled outcomes of systemic restaging with integrated PET-MRI scanners [24] indicate the feasibility of this novel modality for salvage treatment decision-making.

Our study has several limitations. The major limitation is the low number of included studies. All included studies were retrospective and evaluated small samples which makes the risk of bias unavoidable. MRI protocols as well as primary treatment (EBRT and/or BT) differed between studies. This should be highlighted because the use of metallic seeds in brachytherapy might potentially confound the image quality independently from post-radiative tissue changes. The heterogeneity of MRI diagnostic performance between the studies was significant. In particular, almost half of the sample was constituted by cohort staged with T2-WI imaging only. The majority of studies were from high-volume oncological centers with expertise in MRI assessment making our data not transferable to every clinical community. Furthermore, MRI assessment in analyzed studies was based on the index lesion concept similarly to this used in a primary setting. Since radio-recurrent PCa is commonly multifocal, the final MRI utility might be additionally compromised by missing secondary aggressive spots. Finally analyzed data were insufficient to estimate to what extent MRI would change management or benefit survival. The following limitations might potentially prevent drawing any definite conclusions.

## Conclusions

Our systematic review of evidence revealed the true shortage of studies evaluating the utility of MRI in the radio-recurrent setting. We provide the first meta-analysis on the reliability of MRI as a local staging tool after primary radiation with the whole-mount specimen as a reference. The main findings of our study are poor sensitivity and high specificity of MRI when predicting adverse pathology in the post-SRP specimen. It should be, however, emphasized that due to the small sample size and major heterogeneity between the included studies, the results of our meta-analysis bear the inevitable risk of bias and should be interpreted with caution.

## Supplementary Information

Below is the link to the electronic supplementary material.

Supplementary file1 (DOCX 37 KB)

Supplementary file2 (DOCX 41 KB)

Supplementary file3 (DOCX 41 KB)

Supplementary file4 (DOCX 418 KB)

Supplementary file5 (DOCX 39 KB)

## Data Availability

The datasets analyzed during the current study are available from the corresponding author upon reasonable request.

## References

[1] Vora SA, Wong WW, Schild SE, Ezzell GA, Andrews PE, Ferrigni RG (2013). Outcome and toxicity for patients treated with intensity modulated radiation therapy for localized prostate cancer. J Urol.

[2] Potters L, Morgenstern C, Calugaru E, Fearn P, Jassal A, Presser J (2005). 12-year outcomes following permanent prostate brachytherapy in patients with clinically localized prostate cancer. J Urol.

[3] Fendler WP, Calais J, Eiber M, Flavell RR, Mishoe A, Feng FY (2019). Assessment of 68Ga-PSMA-11 PET accuracy in localizing recurrent prostate cancer. JAMA Oncol.

[4] de Rooij M, Hamoen EHJ, Witjes JA, Barentsz JO, Rovers MM (2016). Accuracy of magnetic resonance imaging for local staging of prostate cancer: a diagnostic meta-analysis. Eur Urol.

[5] Coakley FV, Hricak H, Wefer AE, Speight JL, Kurhanewicz J, Roach M (2001). Brachytherapy for prostate cancer: endorectal MR imaging of local treatment-related changes. Radiology.

[6] Quero L, Vercellino L, de Kerviler E, Mongiat-Artus P, Culine S, Merlet P (2015). 18F-Choline PET/CT and prostate MRI for staging patients with biochemical relapse after irradiation for prostate cancer. Clin Nucl Med.

[7] Arumainayagam N, Kumaar S, Ahmed HU, Moore CM, Payne H, Freeman A (2010). Accuracy of multiparametric magnetic resonance imaging in detecting recurrent prostate cancer after radiotherapy. BJU Int.

[8] Ménard C, Iupati D, Publicover J, Lee J, Abed J, O’Leary G (2015). MR-guided prostate biopsy for planning of focal salvage after radiation therapy. Radiology.

[9] Akin O, Gultekin DH, Vargas HA, Zheng J, Moskowitz C, Pei X (2011). Incremental value of diffusion weighted and dynamic contrast enhanced MRI in the detection of locally recurrent prostate cancer after radiation treatment: preliminary results. Eur Radiol.

[10] Zattoni F, Kawashima A, Morlacco A, Davis BJ, Nehra AK, Mynderse LA (2017). Detection of recurrent prostate cancer after primary radiation therapy: an evaluation of the role of multiparametric 3T magnetic resonance imaging with endorectal coil. Pract Radiat Oncol.

[11] Sala E, Eberhardt SC, Akin O, Moskowitz CS, Onyebuchi CN, Kuroiwa K (2006). Endorectal MR imaging before salvage prostatectomy: tumor localization and staging. Radiology.

[12] Kowa JY, Soneji N, Sohaib SA, Mayer E, Hazell S, Butterfield N (2021). Detection and staging of radio-recurrent prostate cancer using multiparametric MRI. Br J Radiol.

[13] Sutani S, Yorozu A, Toya K, Nishiyama T, Ozu C, Yagi Y (2019). Whole-gland salvage treatment for recurrent prostate cancer after initial definitive radiotherapy: a case series of 125I brachytherapy and robot-assisted radical prostatectomy. J Contemp Brachyther.

[14] Bloch BN, Furman-Haran E, Helbich TH, Lenkinski RE, Degani H, Kratzik C (2007). Prostate cancer: accurate determination of extracapsular extension with high-spatial-resolution dynamic contrast-enhanced and T2-weighted MR imaging—initial results. Radiology.

[15] Christophe C, Montagne S, Bourrelier S, Roupret M, Barret E, Rozet F (2020). Prostate cancer local staging using biparametric MRI: assessment and comparison with multiparametric MRI. Eur J Radiol.

[16] Haider MA, Chung P, Sweet J, Toi A, Jhaveri K, Ménard C (2008). Dynamic contrast-enhanced magnetic resonance imaging for localization of recurrent prostate cancer after external beam radiotherapy. Int J Radiat Oncol Biol Phys.

[17] Donati OF, Jung SI, Vargas HA, Gultekin DH, Zheng J, Moskowitz CS (2013). Multiparametric prostate MR imaging with T2-weighted, diffusion-weighted, and dynamic contrast-enhanced sequences: are all pulse sequences necessary to detect locally recurrent prostate cancer after radiation therapy?. Radiology.

[18] Panebianco V, Villeirs G, Weinreb JC, Turkbey BI, Margolis DJ, Richenberg J (2021). Prostate magnetic resonance imaging for local recurrence reporting (PI-RR): International Consensus -based Guidelines on multiparametric magnetic resonance imaging for prostate cancer recurrence after radiation therapy and radical prostatectomy. Eur Urol Oncol.

[19] Pecoraro M, Turkbey B, Purysko AS, Girometti R, Giannarini G, Villeirs G (2022). Diagnostic accuracy and observer agreement of the MRI prostate imaging for recurrence reporting Assessment Score. Radiology.

[20] Heijmink SWTPJ, Fütterer JJ, Hambrock T, Takahashi S, Scheenen TWJ, Huisman HJ (2007). Prostate cancer: body-array versus endorectal coil MR imaging at 3 T—comparison of image quality, localization, and staging performance. Radiology.

[21] Fütterer JJ, Engelbrecht MR, Huisman HJ, Jager GJ, Hulsbergen-van-De-Kaa CA, Witjes JA (2005). Staging prostate cancer with dynamic contrast-enhanced endorectal MR imaging prior to radical prostatectomy: experienced versus less experienced readers. Radiology.

[22] Karzai F, Walker SM, Wilkinson S, Madan RA, Shih JH, Merino MJ (2021). Sequential prostate magnetic resonance imaging in newly diagnosed high-risk prostate cancer treated with neoadjuvant enzalutamide is predictive of therapeutic response. Clin Cancer Res.

[23] Kumar S, Shelley M, Harrison C, Coles B, Wilt TJ, Mason M (2006). Neo-adjuvant and adjuvant hormone therapy for localised and locally advanced prostate cancer. Cochrane Database Syst Rev.

[24] Evangelista L, Zattoni F, Cassarino G, Artioli P, Cecchin D, dal Moro F (2021). PET/MRI in prostate cancer: a systematic review and meta-analysis. Eur J Nucl Med Mol Imaging.

