# Supplementary figures and images for: Magnetic resonance imaging (MRI) for local staging before salvage radical prostatectomy: a meta-analysis
Source: World J Urol. 2023 Apr 5;41(5):1275–84. doi: 10.1007/s00345-023-04383-2 (PMC10188391; doi:10.1007/s00345-023-04383-2)

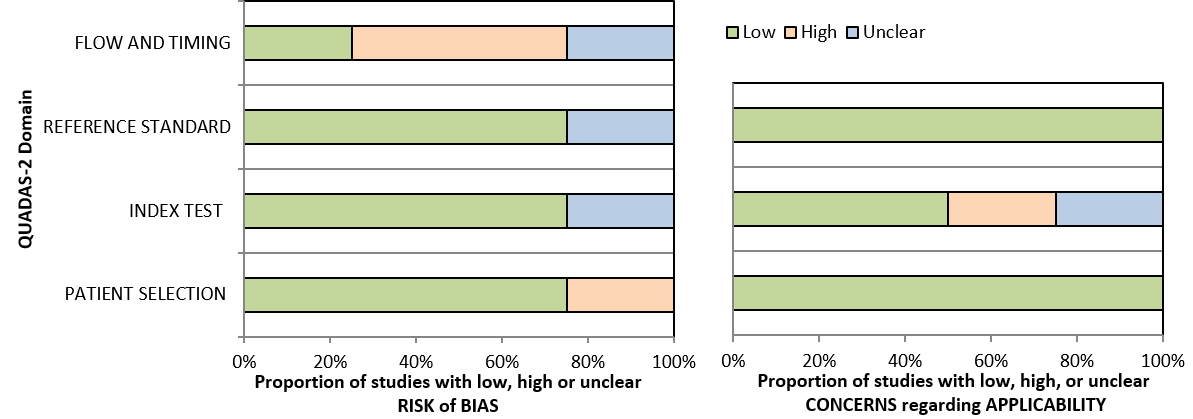


Supplementary Figure 1. Risk of bias and applicability concerns according to QUADAS-2.

Supplement: Supplementary file 1 — Supplementary file1 (DOCX 37 KB) [file 345_2023_4383_MOESM1_ESM.docx]
