# Supplementary material for: Magnetic resonance imaging (MRI) for local staging before salvage radical prostatectomy: a meta-analysis
Source: World J Urol. 2023 Apr 5;41(5):1275–84. doi: 10.1007/s00345-023-04383-2 (PMC10188391; doi:10.1007/s00345-023-04383-2)
Supplement: Supplementary file 4 — Supplementary file4 (DOCX 418 KB) [file 345_2023_4383_MOESM4_ESM.docx]

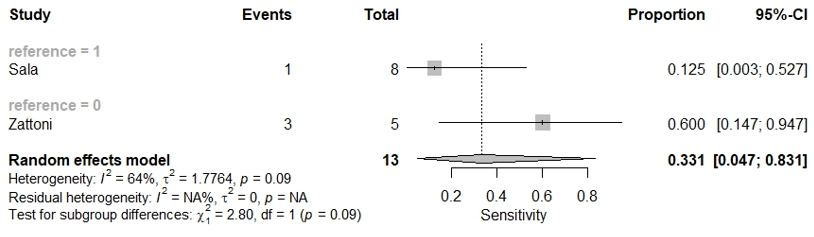

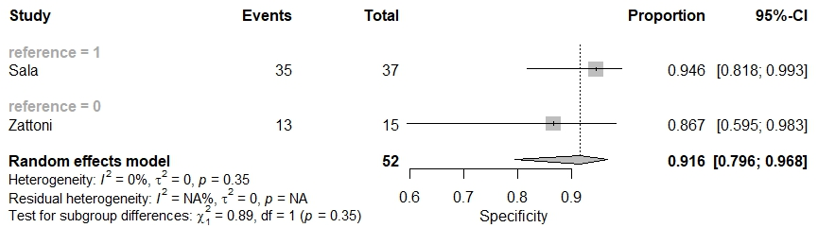


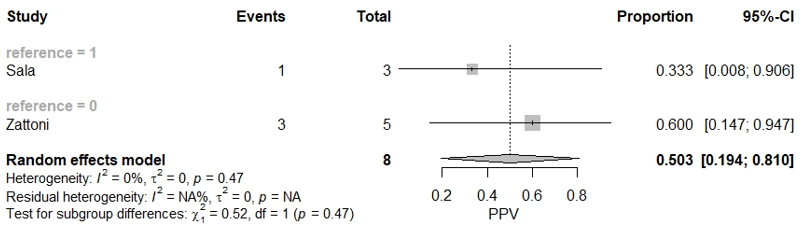


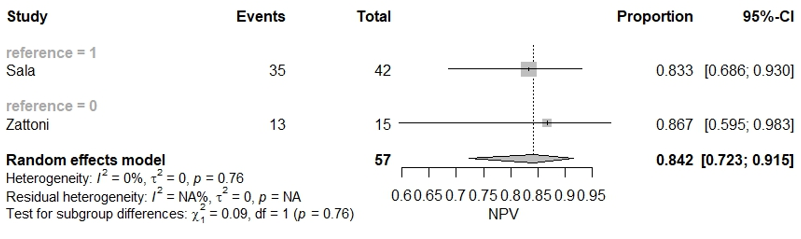


Supplementary figure 4. Forest plots for pooled sensitivity, specificity, PPV and NPV of lymph node invasion detection.
