# Supplementary material for: Magnetic resonance imaging (MRI) for local staging before salvage radical prostatectomy: a meta-analysis
Source: World J Urol. 2023 Apr 5;41(5):1275–84. doi: 10.1007/s00345-023-04383-2 (PMC10188391; doi:10.1007/s00345-023-04383-2)
Supplement: Supplementary file 5 — Supplementary file5 (DOCX 39 KB) [file 345_2023_4383_MOESM5_ESM.docx]

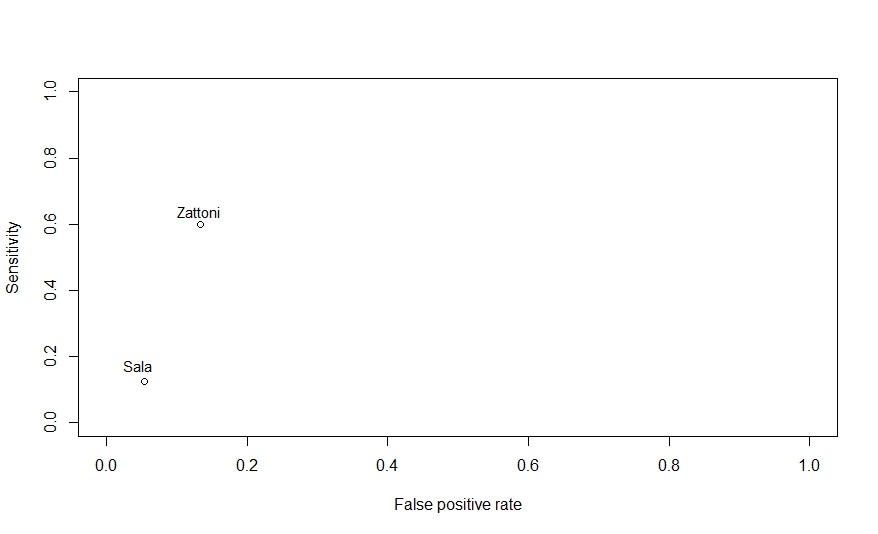


Supplementary figure 5. Dot plot illustrating the association of sensitivity and false positive rate of included studies for detection of lymph node invasion.
